# Relapse in pituitary adenoma after resection

**DOI:** 10.1186/1687-9856-2013-S1-P199

**Published:** 2013-10-03

**Authors:** Nur Rochmah, Frida Soesanti, Aman B Pulungan, Bambang Tridjaja, Jose RL Batubara

**Affiliations:** 1 Department of Child Health Medical School of Airlangga University-Dr Soetomo Hospital Surabaya, Indonesia; 2 Department of Child Health Medical School of University of Indonesia-Ciptomangunkusumo National Hospital Jakarta, Indonesia

**Keywords:** pituitary adenoma, panhypopituitarism, surgery, relapse

## 

Pituitary adenoma in children is rarely reported. Acromegaly is one of clinical manifestation in GH releasing-pituitary adenoma. Recurrence of clinical manifestation after resection must be evaluated for possibility of pituitary adenoma relapse.

N,male,15-yo, came to pediatric endocrinology outpatient clinic with the main complain of acromegaly and decreased of visual field which was getting worse since two weeks before(April11, 2011). He was consulted to ophthalmology and neurosurgery outpatient clinic. MRI with contrast revealed pituitary adenoma. Laboratories results showed TSHS:0.9773(0.35-4.94)uIU/ml, prolactin:0.51(4.04-15.2)ng/ml, testosterone less than 2.50(boys:13-17:28-1110)ng/ml, growth hormone was more than 40,00(>10.0)ng/ml. He was performed transsphenoidal removal cystic tumor. Pathological result showed macroscopic: yellowish cystous mass;0.6x0.4x0.2cm whether microscopic: appropiate to pituitary adenoma, non chromophobe. After surgery, patient was given DDAVP nasal spray 10 microgram/day, L-thyroxin 100 microgram once daily. One year after surgery, patient complaint of acromegaly, decreased visual field, especially in right and left temporal side, cephalgia. On physical examination, body weight was 91.5kg, height was184.5 cm. There was hemianopsia bitemporal. Tanner stage was A2P4G4. MRI with contrast showed pituritary adenoma relapse. Bone age was normal with height percentage based on it is about 96.8%. Tanner Whitehouse showed adult height 186.4cm. Thorax X ray showed heart and lungs were normal. Laboratories results revealed IGF1:1359(237-996)microgram/L, FT4:1(0.89-1.76)ng/dl; TSHS:0.3(0.5-4.94)microIU/ml(12-18yo), testosterone:435.1(28-1110) ng/dl. Working diagnosis was pituitary adenoma relapse post tumor resection, panhypopituitarism, diabetes insipidus. Testosterone 150mg once per month was added. **R**elapse of pituitary adenoma in children must be considered in the recurrence of clinical manifestations.

